# Extracts and Abstracts

**Published:** 1885-10

**Authors:** 


THE *Medical Record* (N. Y., Sept. 19th, 1885,) contains a brief editorial entitled "A Beginning in Bacteriotherapy," in which allusion is made to the fact that certain micro-organisms act destructively upon others. An illustration is presented in a case of pulmonary tuberculosis treated by Dr. Arnaldo Cantani, of Naples, which is described as presenting the various symptoms of the disease in the advanced stage. The experiment consisted in the withdrawal of all medicines and the daily inhalation, by means of an ordinary atomizer, of a mixture of gelatine beef-broth and a pure culture of bacterium-termo. It is stated in the report that an almost immediate improvement occurred, and that in less than a month no bacilli could be found in the sputa, their place being taken by the bacterium-termo; the general condition had greatly improved, and the body-weight increased one and one-half pounds. Apart from the novelty of the experiment, the chief interest consists in results not detailed since the first of June last. We await with interest further report regarding the result of this experiment.

### A NEW REMEDY FOR HICCOUGH.

Dr. O. T. Shultz, of Mount Vernon, Indiana, ("American Practitioner"), relates a case of hiccough of nine days' duration, which yielded, on the morning of the tenth day, to one drop of a one per cent. solution of nitro-glycerine, and repeated in one hour. The remedy was continued at intervals of two hours,—gradually extended. On the twelfth day permanent relief was obtained and treatment discontinued.

